# The Nonbacterial Microbiome: Fungal and Viral Contributions to the Preterm Infant Gut in Health and Disease

**DOI:** 10.3390/microorganisms11040909

**Published:** 2023-03-31

**Authors:** Adam Wilson, Brett Bogie, Hala Chaaban, Kathryn Burge

**Affiliations:** Division of Neonatal-Perinatal Medicine, Department of Pediatrics, University of Oklahoma Health Sciences Center, Oklahoma City, OK 73104, USA

**Keywords:** microbiome, fungi, virus, metagenomics, prematurity, necrotizing enterocolitis

## Abstract

The intestinal microbiome is frequently implicated in necrotizing enterocolitis (NEC) pathogenesis. While no particular organism has been associated with NEC development, a general reduction in bacterial diversity and increase in pathobiont abundance has been noted preceding disease onset. However, nearly all evaluations of the preterm infant microbiome focus exclusively on the bacterial constituents, completely ignoring any fungi, protozoa, archaea, and viruses present. The abundance, diversity, and function of these nonbacterial microbes within the preterm intestinal ecosystem are largely unknown. Here, we review findings on the role of fungi and viruses, including bacteriophages, in preterm intestinal development and neonatal intestinal inflammation, with potential roles in NEC pathogenesis yet to be determined. In addition, we highlight the importance of host and environmental influences, interkingdom interactions, and the role of human milk in shaping fungal and viral abundance, diversity, and function within the preterm intestinal ecosystem.

## 1. Introduction

Necrotizing enterocolitis (NEC) is a devastating neonatal gastrointestinal emergency, affecting 7% of premature and low birthweight (<1500 g) infants [1]. Though the etiology is not completely understood, NEC pathogenesis is multifactorial, encompassing interactions among immature intestinal barrier function, hyperactive immune cells, formula feeding, maternal and neonatal medications, and dysbiosis [2,3]. Infants with NEC suffer from severe inflammation of the distal intestine, and despite surgical resection of the affected bowel in 35% of cases, a dearth of treatment options beyond surgical and supportive care results in a mortality rate in the United States approaching 30% [1,4].

The role of dysbiosis, a condition of limited species and functional richness within the host microbiome [5], in NEC pathogenesis has been a recent focus of investigation [2,6,7]. A developmentally appropriate microbiome, encompassing all microbes, microbial genes (metagenome) and proteins (metaproteome), and microenvironmental constituents of the intestinal lumen [8], is thought to be critical in prompting maturation of the infant intestinal epithelium and immune system [9]. Additionally, a healthy microbiome provides energy for both adjacent microbes and the host [10], as well as structure and defense for the intestinal epithelium [11,12], thereby helping to protect against NEC [13]. The importance of the microbiome to NEC development is highlighted by the finding that, while a single pathogen is not responsible for NEC development [6], germ-free animal models of NEC do not develop the disease [14,15,16].

Intestinal colonization of the preterm infant, with some debate [17,18], begins at birth [12,19] and progresses generally from aerobic Bacilli to Gammaproteobacteria to anaerobic Clostridia as the intestine matures [20]. However, this progressive colonization in preterm infants results in a microbial composition lower in species diversity and richness than that of term infants, often with a predominance of pathogenic bacteria capable of triggering intestinal inflammation [21]. A number of factors, including antibiotic utilization [19,22,23,24,25], mode of delivery [26,27,28,29,30], antacid use [31,32,33], mode of feeding [34,35,36,37,38,39] and maternal diet [40], and individual patterns of defensin and lysozyme secretion within the intestinal epithelium [41,42], can influence the microbial colonization of the malleable preterm infant gut. In preterm infants developing NEC, however, depletion of species and functional diversity is especially evident [43,44,45], with reductions in Bifidobacteria, Bacteroidetes, and Firmicutes, and parallel increases in Proteobacteria and Actinobacteria. These bacterial shifts often precede diagnosis of the disease [46], potentially indicating a causal role for these microbes in NEC development [7,26,44,46,47,48,49,50].

Due to the relative ease of identification and abundance, bacteria (eubacteria) are typically the only component of the microbiome reported in studies associating dysbiotic changes with preterm NEC development, and the terms microbiota and microbiome are often incorrectly and confusingly reported as only the bacterial components of each. However, microbiota comprising the human microbiome also include eukaryotic fungi and protozoa, archae, and viruses [13,51,52], and omission of these critical microbial components of the preterm intestinal community when reporting microbiome outcomes paints an incomplete and biased picture of community roles and health [53,54]. Very few studies report nonbacterial microbial components of the preterm infant microbiome, with the majority indicating an association with NEC pathogenesis [54,55,56,57,58,59]. However, nonbacterial microbiota are healthy components of term infant [60], as well as adult [51], microbial communities. Thus, the apparent skew in reporting toward pathogenicity likely represents a substantial gap in nonbacterial knowledge rather than a uniquely negative role for these organisms in host health [61,62]. Here, we briefly summarize current high-throughput sequencing and bioinformatics methods used to identify bacterial and nonbacterial constituents of the preterm microbiome. In addition, we expand upon the potential role of nonbacterial microbiome members, namely fungi and viruses, in preterm infant health and NEC susceptibility. Finally, we discuss physiological and environmental factors interfacing with the immature and hyperinflammatory preterm infant gut, the latter reviewed extensively elsewhere (e.g., [63,64]), that may influence the colonization, growth, and function of these organisms.

## 2. Gene Amplicon and Metagenomic Sequencing

By avoiding the requirement for culturing organisms, gene amplicon and metagenomic sequencing methods have become high-throughput. Gene amplicon sequencing, still the most commonly utilized technique for describing microbiome composition and function, can distinguish prokaryotic (16S rRNA [ribonucleic acid]) or eukaryotic (18S rRNA or internal transcribed spacer [ITS] regions of the rRNA) taxa through targeted polymerase chain reaction (PCR) amplification of hypervariable regions of a widely expressed gene [65]. The high conservation of these selected genes results in widespread inclusion of analyzed taxa, while the variable regions of this gene allow for discrimination among organisms, typically to the level of genera. Metagenomics, the shotgun analysis of entire genomes in a sample, has further improved the ability to characterize environmental and clinical samples of microbial communities [65]. This technique, often referred to as whole-genome shotgun metagenomic sequencing (WGS), fragments total sample deoxynucleic acid (DNA) and aligns random sequences with those annotated in prokaryotic and eukaryotic genome databases [65]. The focus on all protein-coding sequences with WGS rather than amplification of a single sequence allows for improved functional predictions for the microbiome, as well as the potential for greater resolution and accuracy in identifying lower frequency eukaryotic and viral microbiome constituents [65,66]. Importantly, both sequencing methods can be used to characterize functional traits, such as antimicrobial resistance (AMR) genes [67]. A further method, meta-transcriptomics, is often used to characterize RNA viruses of the microbiome through sequencing of only the non-ribosomal RNA within a sample [68], though RNA viruses are subject to instability [69].

16S rRNA sequences are referenced to rRNA databases (e.g., Greengenes, SILVA) [70]. Gene amplicons are further analyzed through methods clustering similar sequences into operational taxonomic units (OTUs; e.g., Quantitative Insights into Microbial Ecology 2 [QIIME 2]) or reducing noise among amplicon sequence variants (ASVs) prior to clustering (e.g., DADA2), thereby enhancing sequence resolution [66,71,72]. Sequences derived from WGS, on the other hand, are mapped to protein-coding genomic databases (e.g., RefSeq, GenBank) to infer functional roles for microbiota. The cost of WGS, the inability of WGS to distinguish between sequences derived from living or deceased organisms, and incomplete reference databases often limit the utility of this method in favor of 16S rRNA amplicon sequencing [72].

WGS is typically run on 200–300 base pair (bp) short-read platforms (e.g., Illumina) or on 500–4000 bp long-read platforms (e.g., PacBio or Oxford Nanopore Technology [ONT]). Short-read methods have been more popular to date as the cost is controlled through producing billions of short reads able to discriminate only to the level of genera [71]. Second-generation sequencing methods (e.g., Ion Torrent), although providing longer reads and greater discrimination, have largely proven unpopular due to prohibitive cost. However, third-generation long-read sequencing platforms (e.g., MinION and PacBio) are transforming metagenomic sequencing through longer read depth and decreased costs [71]. In particular, the ONT MinION provides increased system accessibility and portability, with the potential for real-time screening in clinical or field settings [73]. The MinION is capable of resolving taxa to the level of species, characterizing species abundance, and identifying functional gene signatures. Likely, a compromise among sequencing depth, number of reads, and novel bioinformatic approaches for sequence reference alignments will produce the most robust representation of the preterm human microbiome.

## 3. Preterm Infant Mycobiome

In contrast to that of bacteria, the influence of fungal microbiome constituents (mycobiota) on preterm infant health and disease has been relatively unexplored (Table 1). While there are far fewer fungal than bacterial genomes within the gut, a fungal cell is roughly 100-fold larger in volume than a bacterial cell, suggesting fungal constituents of the microbiome contribute substantial biomass and are underappreciated as key members of the gut microbiota [74]. However, difficulty establishing reference sequences and the inherent complexity of sequencing low genome species have contributed to a lag in fungal studies [75,76].

In some respects, fungal seeding of the preterm infant gut appears to follow a similar pattern to that of bacterial seeding, with maternal transmission during birth responsible for many of the first colonizers [77,78], and environmental and biological factors (e.g., gestational age, sex, modes of delivery and feeding, neonatal intensive care unit [NICU] practices, and medication use) dictating subsequent dynamics [74,75,79,80,81]. However, in contrast to the relative explosion in diversity and number of bacteria postnatally, infant fungal communities progress in complexity while remaining relatively minor, by abundance, components of the microbiome [80,82]. In addition, the mycobiome is characterized by greater intra- and interindividual variation compared with the bacterial microbiome [83]. The term infant gut is most commonly colonized by *Saccharomyces*, *Candida*, and *Malassezia* spp. [80]. Preterm infants harbor a reduced diversity of fungi compared with full-term infants, and often, a single species of *Candida* dominates the preterm mycobiome up to six months of age [57]. Of the initial *Candida* colonizers, *C. albicans* and *C. parapsilosis*, both opportunistic pathogens in hospital settings and commensal species of the adult gastrointestinal tract, are frequent and persistent preterm microbiome members [58,84]. *Aspergillus*, *Davidiella*, *Debaryomyces penicillium*, *Saccharomyces*, and among extremely low birth weight (<1000 g) infants, *Cladosporium* and *Cryptococcus*, have also been identified [79,85]. The less diverse fungal colonization, especially in combination with reduced bacterial diversity [86], may play a critical role in successive microbiota colonization and, thus, mature gut microbiome community structure among preterm infants [87].

Fungi in the gut are present as unicellular yeast, pseudohyphae, and filamentous hyphae; they alter gene expression profiles and virulence factor production when transitioning between morphologies, and provide unique metabolic capabilities to host digestion [82,83]. While the functional role of the mycobiome in preterm infants has not been well-studied, intra- and interkingdom interactions are known to influence overall gut microbiome community function [88]. For example, preterm infant colonization with *S. boulardii* is thought to regulate the growth and invasiveness of *Candida* [75]. Fungal-bacterial interactions drive gut microbial community structure through competition for resources and production of antimicrobial peptides [89,90,91]. Additionally, mutually compatible fungi and bacteria can form polymicrobial biofilms, producing, at times, additive virulence [83]. Evidence of differences in gut interkingdom relationships is apparent between preterm and term infants as early as the first-pass meconium, where the relative presence of *Methylobacterium*, *Bacteroides*, and *Stereum*, and absence of Actinobacteria, Blasidiomycota, and Bacteroidetes, can differentiate preterm from term infants [60]. Growth of the opportunistic *Candida* during early postnatal life is thought to expand in concert with, and as a direct result of, shifts in bacteria from aerobic to anaerobic species [92], while *Candida* overgrowth can promote *Enterococcus faecalis* populations and prevent colonization by *Lactobacillus* [93] and several additional bacterial genera [94]. In addition, Rao et al. performed multi-kingdom SpikeSeq to evaluate both absolute and relative longitudinal composition of bacteria and fungi within the preterm gut, finding a negative correlation with fungal and bacterial loads [94]. Interestingly, *Staphylococcus* appeared to inhibit growth of *Candida*, while *Candida* inhibited growth of *Klebsiella* and *Escherichia*, highlighting the diverse and integrated relationships among gut community members.

As with bacteria, specific colonization patterns of fungal organisms have been linked with infant health outcomes. Infant *Candida* and *Rhodotorula* colonization, for example, has been associated with asthma and atopy [95], while *C. parapsilosis* is frequently isolated from infants in the NICU [96]. Some fungal colonization is a direct result of maternal disease. For instance, *Candida* has been demonstrated to pass vertically to infants from mothers with symptomatic and asymptomatic mammary candidiasis [97]. In addition, the first-pass meconium of preterm infants exposed to perinatal antibiotics is more likely to contain fungal organisms, and specifically *Candida*, compared with that of term infants. As *Candida* has been detected in the amniotic fluid [98] and associated with chorioamnionitis [99], the correlation of this fungus in preterm first-pass meconium may indicate a potential role for maternal fungal infection or dysbiosis in preterm birth [60]. While many fungi are clear opportunistic human pathogens, others, such as *S. boulardii*, have been shown to benefit the adult host through amelioration of gastrointestinal symptoms [100,101], potentially through upregulated intestinal IgA production [102]. While many of the potential benefits of gut fungal colonization have not been explored in the preterm population, a positive association between fungal load and child growth has been noted. Randomized controlled trials supplemented preterm infants with *S. boulardii* and evaluated infant growth. Interestingly, *S. boulardii*-supplemented infants experienced significantly greater weight gain compared to control [103,104], potentially through modulation of the growth hormone axis [105]. Thus, the roles of fungi in the preterm gut appear multifaceted and dependent upon environmental influences.

**Table 1:** Overview of key preterm infant gut fungal sequencing studies.

Authors/Year	Study Population	Samples and Methods	Bioinformatics	Key Findings
Beck et al., 2022 [106]	Preterm infants (<32 wk) with Infloran (*n* = 24), Labinic (*n* = 71), or without (*n* = 28) probiotics, collected between 0 and 120 DOL	Metagenomic shotgun sequencing of stool	MetaPhlAn v.2.0	11 fungal species detected; *C. albicans* and *C. glabrata* most prevalent
Bender et al., 2021 [107]	Preterm infants with (*n* = 7) and without (*n* = 18) antibiotics	ITS amplicon sequencing of stool	QIIME, DADA2	Limited evidence of fungal involvement in overall microbiome; no antibiotic effect on fungal colonization
Bliss et al., 2008 [78]	VLBW (<1500 g) infants with (*n* = 46) and without (*n* = 30) maternal *C. albicans* colonization	DNA fingerprinting of stool	N/A	Vertical and horizontal transmission of *C. albicans* occurs in VLBW infants
Henderickx et al., 2022 [79]	Extremely preterm (*n* = 18; 24–27 wk), very preterm (*n* = 15; 28–31 wk), late preterm (*n* = 17; 32–36 wk), and term (*n* = 6; 37–40 wk) infants	ITS2 amplicon sequencing of stool	QIIME 2, DADA2, and UNITE	*Candida* spp. most abundant and ↑ with GA; *Candida* spp. ↑ with vaginal delivery, while *Malassezia* spp. ↑ with C-section; *C. albicans* most abundant in preterm infants
Huang et al., 2022 [108]	NICU infants with clinically diagnosed fungal infection following antibiotic treatment (*n* = 3), infants following antibiotic treatment without fungal infection (*n* = 5), and controls (no antibiotics or fungal infection; *n* = 4)	Stool culture	N/A	Fungal infection associated with ↓ in *Clostridium* spp., *Bacteroides* spp., and intestinal IgA, and ↑ in *Enterococcus* spp.
James et al., 2020 [57]	Preterm infants (*n* = 11; 25 to 36 wk)	ITS1 amplicon sequencing of stool	USEARCH, QIIME 2, UNOISE3, and UNITE	*C. parapsilosis* most common, esp. infants < 31 wk; GA/diet influence fungi
Kaufman et al., 2006 [84]	ELBW infants (*n* = 50; <1000 g)	Stool culture	N/A	*C. albicans* most likely to colonize > 1 body location, often within 2 wk of birth
LaTuga et al., 2011 [85]	ELBW infants (*n* = 11; <1000 g)	ITS3 amplicon sequencing of stool	PyroTagger, Mothur, BLAST	Despite nystatin treatment, *Candida* spp. still present
Leibovitz et al., 2013 [109]	VLBW infants (*n* = 118; <1500 g)	Stool culture	N/A	*C. albicans* and *C. parapsilosis* most common, esp. within 1st 14 DOL
Mendiratta et al., 2006 [110]	Preterm VLBW (*n* = 103; <1500 g) and term (*n* = 100) infants	Stool culture	N/A	*C. albicans* colonization is common and associated with antibiotic use and maternal colonization
Olm et al., 2019 [111]	Preterm infants with (*n* = 34) and without (*n* = 126) NEC	Genome-resolved metagenomics of stool	IDBA-UD; EukRep-based pipeline; BLAST	Fungal colonization highest in 1st wks of life and with antibiotic use
Rao et al., 2021 [94]	Preterm infants (*n* = 178; <33 wk)	Multi-kingdom SpikeSeq ITS1 amplicon sequencing of stool	SILVA, UNITE	Inverse correlation of bacterial and fungal load in preterm gut
Stewart et al., 2013 [54]	Preterm infants (*n* = 32; <32 wks)	28S rRNA DGGE amplicon sequencing of stool	BLASTn	Universal fluconazole use and no viable fungi; *Candida* spp. most abundant
West et al., 2021 [58]	Preterm infants (*n* = 163), collected between 5 and 121 DOL	Genome-resolved metagenomics of stool	IBDA-UD, EukRep, CONCOCT, BUSCO-trained GeneMark-ES and AUGUSTUS on MAKER pipeline	Antifungal resistance genes are present in hospitalized infants; Community interaction of *C. parapsilosis* and *Enterococcus faecalis* influences respective gene and protein expression
Willis et al., 2019 [60]	Preterm (*n* = 30; 23–32 wk, <1500 g) and term (*n* = 16; 37–41 wk) infants	ITS2 amplicon sequencing and culture of first-pass meconium	QIIME; UNITE	Fungus culturable in first-pass meconium; ↑ Saccharomycetes (esp. *Candida*) in some preterms

DOL, days of life; MetaPhlAn, metagenomic phylogenetic analysis; ITS, internal transcribed spacer; QIIME, quantitative insights into microbial ecology; VLBW, very low birthweight; DADA2, divisive amplicon denoising algorithm; NICU, neonatal intensive care unit; GA, gestational age; ELBW, extremely low birth weight; BLAST, basic local alignment search tool; CONCOCT, clustering contigs on coverage and composition; BUSCO, benchmarking universal single-copy orthologs; DGGE, denaturing gradient gel electrophoresis; BLASTn, nucleotide BLAST.

## 4. Preterm Invasive Fungal Infection (IFI)

Invasive fungal infection (IFI), most often involving epithelial translocation of *Candida*, occurs in preterm infants when dysbiosis, compromised intestinal barrier function, or underdeveloped or weakened immune defenses allow for overgrowth and invasion of commensal [83] or environmental fungi [112]. Due to increased risk and susceptibility to infection, preterm infants are frequently exposed to broad-spectrum antibiotic usage [113]. Diminished bacterial biomass in the gut then allows for fungal expansion and overgrowth, with a fraction of these fungal colonization events resulting in infection [114,115,116]. Interplay among the preterm immature immune system, underdeveloped intestine, and dysbiosis, along with environmental risk factors such as total parenteral nutrition, invasive catheters and endotracheal tubes, and medications [75,79,83,112,117,118] results in systemic, invasive candidiasis in 10% of preterm infants, with a mortality rate approaching 20% [119,120] and a host of associated long-term morbidities [115]. While fungal infections are thought to be an extraordinarily rare cause of preterm NEC [121,122,123], the weakened intestinal barrier of an infant with NEC may allow for *Candida* coinfection [124,125,126], resulting in, for example, fungal sepsis following surgical NEC treatment [127].

Prophylactic antifungals, including nystatin and fluconazole, are frequently used in the NICU in an attempt to reduce the high rates of invasive candidiasis among preterm, extremely low birthweight (ELBW) infants [75,116,128,129]. Fluconazole, in particular, shows efficacy against *Candida* overgrowth in ELBW infants [84,130,131,132]. The use of prophylactic probiotics, including not only the bacteria *Lactobacillus reuteri*, *L. casei*, *L rhamnosus*, *L. acidophilus*, *Streptococcus thermophilus*, *Bifidobacterium longum*, *B. bifidum*, and *B. lactis*, but also the fungus *Saccharomyces boulardii*, have also been used to prevent IFI [130,133,134], with variable efficacy [75]. Though fungal overgrowth is not often a direct cause of significant intestinal inflammation in preterm neonates, factors influencing fungal-intestinal interactions may play an indirect role in NEC pathogenesis [135]. Thus, acknowledging and addressing the ways in which fungi may contribute to intestinal health and disease are paramount in improving preterm morbidity and mortality.

## 5. Factors Influencing Fungal Colonization and Invasion within the Preterm Gut

### 5.1. Localized Immune Response

The relationship of commensal or pathogenic gut fungi, in all morphologies [136], with the host immune system in the gut is complex; however, recent studies are advancing knowledge in this field. Host sensing of fungal colonization initiates with innate immune receptor-binding of fungal pattern recognition receptor (PRR) ligands [74], generally keeping fungal overgrowth in check. Membrane-bound C-type lectin receptors (CLR), TLR, or scavenger receptor family members induce neutrophil, monocyte/macrophage, and dendritic cell phagocytosis [137], reactive oxygen species (ROS) production, activation of the pro-inflammatory transcription factor, nuclear factor κB (NF-κB) [74], and secretion of the mature IL-1β cytokine [137]. Soluble receptors induce fungal opsonization and antimicrobial complement (e.g., C3, C5a, factor H, factor I; [138,139,140,141]) activation [74]. If innate immune defense is insufficient, fungi can become invasive, actively penetrating host tissue with hyphae [83], inducing large secretions of IL-8 [142], and transcellularly crossing the intestinal barrier through necrotic tissue [143]. High fungal burdens or invasion results in an adaptive immune response [83,137]. Dendritic cells, activated through PRR-binding, present antigens to CD4^+^ T helper (Th) cells. Expansion of Th17 and Th1 cells, and their respective signature cytokines, IL-17 (Th17), IL-22 (Th17), and IFNγ (Th1), aids in the containment of invasive fungi [52,137]. Finally, hyphae induce B cell production of secretory IgA (sIgA), limiting fungal virulence [144,145].

In healthy adults, fungal interaction with the intestinal epithelium can elicit protective immune responses. For example, mucosa-associated fungi (e.g., *Candida*, *Saccharomycopsis*, and *Saccharomyces*) induce intestinal production of IL-17A, IL-17F, and IL-22, as well as transcriptional upregulation of genes associated with immune response and cell proliferation. Intestinal IL-22 production improved gut barrier permeability and protected against bacterial infection- and antibiotic-induced intestinal injury [146]. Fungal cell wall components, such as mannans, induce innate and adaptive immunity through binding of PRRs [136,147]. Jiang et al. demonstrated mono-colonization with either *C. albicans* or *S. cerevisiae*, or an equivalent volume of mannans, recapitulating the protective effects of commensal bacteria against colitis and viral infection in a TLR4-dependent manner [148]. Interestingly, these protective responses are driven both by direct fungal-host interactions and by indirect influences on the bacterial microbiome composition, in many cases allowing or encouraging fungal colonization but inhibiting fungal invasion [149], similar to the host response to fluconazole treatment [150].

A number of developmental and environmental factors prevent an effective physiological response to fungal overgrowth and infection in preterm infants, potentially initiating or compounding intestinal inflammation associated with NEC. Low levels of mucin production in preterm infants [151,152] enable extensive fungal hyphae contact with the mucosa epithelium [83]. Antimicrobial peptide production is reduced in premature infants [153], and neutrophil numbers and function (e.g., neutrophil extracellular trap [NET] formation [154]) are inhibited compared to term infants or adults [155]. Preterm monocytes and macrophages likely respond to CLR dectin-1 and -2 activation [156], but may do so in a hyperinflammatory manner [157]. While some PRRs to *Candida* ligands may be functionally immature in preterm infants [137], others, such as TLR4, are highly expressed and hyperreactive at baseline [152], and additional activation through fungal interactions may spur ‘runaway’ inflammation typical of NEC [158]. Production of intestinal sIgA in preterm infants is negligible until the third postnatal week [159]. While levels of sIgA are high in preterm colostrum and transitional mother’s own milk (MOM), only 40–60% of very low birthweight (VLBW) infants receive MOM in lieu of donor human milk (HM) or infant formula [160], the latter of which lack substantial levels of sIgA [159].

Production of IL-22 is potentially the most significant immune difference between preterm infant fungal invasion and that of a healthy adult. IL-22 broadly restrains intestinal inflammation and clears intestinal pathogens in adults, including fungal [74], through generation of antimicrobial proteins (e.g., β-defensins, S100 proteins) [161]. In addition, IL-22 spurs intestinal regeneration following injury through enhanced epithelial proliferation [161]. Premature infants appear to produce very low levels of IL-22 at baseline, potentially due to a lack of intestinal commensal stimulation of IL-22-producing cells, and are unable to efficiently upregulate IL-22 production in the setting of intestinal microbial inflammation [161]. Importantly, exogenous recombinant IL-22 can ameliorate NEC inflammation and injury independent of microbiome composition changes in a neonatal mouse model of the disease [161]. In the preterm infant, hyperactivated TLR4 signaling skews Th cell differentiation toward that of Th17 rather than T regulatory cells [162]. Given γδ T cells are also capable of IL-17 production early in gestation [163], the cumulative high secretion of IL-17 without the regenerative potential of IL-22 likely produces an inflammatory environment conducive to tissue destruction. In addition to these developmental limitations, fungi are more resilient than bacteria to suboptimal environmental features common in the preterm population, including deviations in pH, temperatures, or CO_2_ levels [83], resulting in fungal infections that are very difficult to clear.

### 5.2. Antifungal Medication

Antifungal prophylaxis is commonly prescribed for infants at risk of, or diagnosed with, invasive fungal infections [129,150]. Interestingly, treatment with fluconazole or nystatin typically does not inhibit fungal colonization, but rather alters gut fungal composition and prevents fungal invasion of the gastrointestinal mucosa [82,85]. Antifungal treatment is protective against fungal-driven intestinal disease in mouse models [164], but exacerbates intestinal inflammation in conditions lacking frank fungal involvement [165]. In addition, antifungals are known to alter gut bacterial composition [165] and have direct antibacterial properties [166]. For example, adult fluconazole treatment reduces populations of *Lactobacillus* [165], a genus of bacteria whose relative absence in NEC has been associated with increased intestinal inflammation, reduced tight junction protein expression and barrier integrity, and reduced bacterial diversity within the infant gut microbiome [167]. Taken together, while the effects of antifungal treatment on preterm intestinal health, at homeostasis or during inflammation, have not yet been elucidated, prolonged or excessive use of antifungals, much as with extended use of broad-spectrum antibiotics [25,168,169], may directly or indirectly contribute to intestinal inflammatory disease.

### 5.3. Broad-Spectrum Antibiotics

A common observation is that bacterial dysbiosis often goes hand-in-hand with invasive fungal infection [75]. The widespread use of broad-spectrum antibiotics [170,171], including those frequently used within the NICU (e.g., vancomycin and gentamicin [172]), drastically alters both bacterial and fungal communities of the infant gut, inducing an increase in Proteobacteria, a reduction in *Bifidobacteria* [66], and allowing opportunistic pathogens, such as *Candida* spp., to proliferate [75]. Further, cephalosporin use in preterm infants has been associated with increased risk for invasive, systemic candidiasis [173]. Increases in fungal colonization and overgrowth, including a persistent increase in fungal diversity and richness [174], have been demonstrated following long-term antibiotic use [175], likely due to altered intestinal metabolites promoting *Candida* expansion [176]. Fungal overgrowth, in fact, may be more related to alterations in the bacterial microbiome following antibiotic use than with factors directly associated with fungal or immune cells of the gut.

### 5.4. Human Milk Feeding

Infant feeding mode directly influences mycobiome seeding and maintenance [177]. Breastfeeding is understood to reduce intestinal infection rates among preterm infants [178,179], but the role of HM fungi or the effects of gut fungal colonization on preterm intestinal health have not been elucidated. Studies estimate 20 to 80% of HM samples contain viable fungi [180,181], though neither the origin of these organisms nor their effect on the infant intestine is clear. *Candida*, *Alternaria*, *Saccharomyces*, *Malassezia*, and *Rhodotorula* spp. represent the most common fungi in HM [180,182], many of which may be transferred from the mother’s skin.

Breastfeeding is also a source of several unique bioactive factors either not present, or present in lower quantities, in infant formula. HM oligosaccharides (HMOs), complex glycan prebiotics metabolizable by Bifidobacteria and Bacteroides [135,183], have been associated with reduced rates of NEC [184,185]. In addition to inducing favorable effects on the infant bacterial microbiome [186,187], HMOs have been demonstrated to inhibit fungal-host cell interactions, reduce hyphal morphogenesis, and decrease *C. albicans* invasion of preterm intestinal epithelial cells in vitro [135], potentially indicating these abundant compounds may also protect against neonatal invasive fungal infections. Levels of the HMO, disialyllacto-N-tetraose (DSLNT), are significantly lower in MOM of NEC infants compared with age-matched controls [188]. Interestingly, DSLNT levels are negatively associated with HM fungal load [180], but whether this relationship is predicated upon fungal utilization of DSLNT or other factors, including the bacterial composition of MOM and the infant microbiome, is unclear. Another component of HM, lactoferrin, has been demonstrated to have antimicrobial activity against viral infection, late-onset sepsis, NEC, and systemic fungal infection in preterm infants [189,190]. In addition, lactoferrin encourages colonization of a healthy preterm microbiome, promoting levels of *Bifidobacterium* and *Lactobacillus* within the gut. Due to high structural homology, both bovine and human lactoferrin prophylaxis are effective in checking fungal invasiveness through disruption of cell wall integrity, while not affecting fungal colonization rates [191]. Unfortunately, these and many other benefits provided through HM are not available to a significant number of preterm infants, as substantial barriers to breastfeeding in the NICU result in low rates of MOM feeding among preterm infants [192].

## 6. Fungal Prevention of Intestinal Inflammation

Very few studies have evaluated the potential of fungi, or their products, to ameliorate intestinal inflammation, and fewer still in the context of preterm infants. Takata et al. demonstrated oral supplementation with *C. kefyr* protects against murine colitis-associated intestinal injury through alterations in gut bacterial composition, increases in T regulatory cells, and decreases in Th17 cells of the lamina propria [193]. Zhang et al. evaluated the ability of the *S. cerevisiae* cell wall component, β-glucan, to protect against murine NEC. Pretreatment with β-glucan for 7 d reduced gut permeability, ameliorated intestinal inflammation, and induced beneficial changes to the bacterial microbiome via modulation of the TLR4/NF-κB pathway [194]. These results, in combination with an earlier study indicating β-glucan reduces inflammation in a neonatal rat NEC model [195], show promise in the ability of fungal components to constrain intestinal inflammation in the preterm infant population.

## 7. Preterm Infant Virome

Of the relatively common constituents of the preterm microbiome, likely the least is known about viruses (Table 2). Viruses are ubiquitous, accounting for the bulk of all genetic material on Earth, but studies have largely focused only on those considered important in disease transmission [68]. Understanding of the importance of viral microbiome contributions (virome) to preterm infant health and disease is lacking. Temporal changes in preterm postnatal viral signatures are dynamic, and less than 1% of viruses have been characterized [196], deeming many identified sequences novel [197]. Given the infant viral biomass is a significant 10^8^ virus-like particles (VLPs)/g stool within a single week of birth [197], methodological challenges will need to be overcome to explore the likely vital role of viruses in the preterm infant gut microbial ecosystem.

Both DNA and RNA viruses, single- and double-stranded, have been identified in the gut of preterm infants, with the former typically consisting of bacteria-infecting bacteriophages (phages) [198] and the latter more associated with maternal diet [197]. Eukaryotic viruses, those capable of infecting human cells, are far less common than prokaryotic viruses [199], such as phages. Viruses have generally not been identified in healthy amniotic fluid [200] or infant meconium [197,201], but are often present within days of birth, most likely via nosocomial, dietary, or environmental sources, rather than direct maternal seeding [201]. However, the earliest viral colonizers may represent indirect maternal vertical transmission of prophages activated by bacteria common in the maternal gut or HM, such as Bifidobacteria, *Lactobacillus*, and *Streptococcus* [202,203,204,205]. As with bacterial colonization, the gut virome is heavily influenced by birth and feeding mode, with vaginal delivery associating with a more diverse viral and bacteriophage composition [206,207]. Interestingly, higher maternal vaginal eukaryotic viral diversity is associated with preterm birth, potentially implying the unique physiology of pregnancy may regulate the virome likely to be transmitted to the infant through birth [208]. Postnatal viral diversity is initially low, especially in comparison to that of adults, but slowly increases in complexity in line with bacterial diversity [197]. Specifically, eukaryotic viruses are found in low abundance and richness at birth and increase over time [209,210,211], with astrovirus (ASV) becoming the most commonly detected enteric virus among preterm infants [55], while phages, particularly, *Caudovirales* [212], are highly abundant at birth and decrease as the bacterial microbiome stabilizes around 2 y [209]. However, infant viral diversity is likely significantly underestimated due to the sheer number of unidentified viral sequences. Significant variability in preterm viral signatures exists among individuals, but, in comparison with the mycobiome, intraindividual variability is diminished [56].

Viral, particularly phage, interactions with colonizing bacteria in the preterm intestine are likely critical in shaping development of the infant microbiome and host mucosal immunity [213,214,215]. Phages can influence bacterial metabolism and growth, as well as promote horizontal transfer of AMR genes [199]. Lytic phages infect bacterial cells, replicate, and then spread through cell lysis, while temperate phages integrate into the bacterial DNA (prophage) and remain quiescent, only lysing the bacterial host upon exposure to an environmental signal [210], such as oxidative stress or antibiotic use [199]. Thus, phages impact bacterial seeding and succession in the gut, inhibiting prolonged blooms of dominant bacteria through infection by the corresponding phage [197]. The tracking of phages to dominant bacteria likely explains much of the significant and early variability in the infant gut, as bacterial blooms progress to a more stable gut environment [216,217]. Interestingly, most VLPs in the infant gut appear to represent temperate prophages, derived from initial bacterial colonizers of the gut microbiome [210]. While many detected viruses remain unclassified, the bacteriophage families *Myoviridae*, *Podoviridae*, and *Siphoviridae* are well-represented in the preterm gut [56].

Vertical transmission of the maternal virome, particularly phages, is thought to occur, in part, through HM feeding [205]. Bifidobacteria bacteriophages, for example, have been demonstrated in HM [203], and HM feeding enhances growth of both beneficial bacteria and their respective associated phages [210]. Formula feeding has been associated with higher infant eukaryotic viral loads in the stool [210], while HM protects against eukaryotic viruses [198]. One distinct exception to HM protection from eukaryotic viruses is cytomegalovirus (CMV), which can readily pass through HM to the infant with dire consequences [218]. Pasteurization of HM is effective at preventing maternal transmission of CMV to neonates [219]. HM immune cells identify and respond to viral replication [220], maternal antibodies can directly bind VLPs [221], lactoferrin coats VLPs and prevents host cellular uptake [190,222,223], and HMOs serve as decoys for viruses attempting to attach to the mucosa [224,225].

**Table 2:** Overview of key preterm infant gut viral sequencing studies.

Authors/Year	Study Population	Samples and Methods	Bioinformatics	Key Findings
Beck et al., 2022 [106]	Preterm infants (<32 wk) with Infloran (*n* = 24), Labinic (*n* = 71), or without (*n* = 28) probiotics, collected between 0 and 120 DOL	Metagenomic shotgun sequencing of stool (DNA viruses only)	VirMap v.1.0	CMV (7 infants) and betapolyomavirus (1 infant) detected
Brazier et al., 2017 [201]	Infants (*n* = 15; 3 < 37 wk) born in unindustrialized Africa	Multiplex real-time PCR of meconium and stool (RNA viruses only)	Geneious R7 and MEGA7	RVA, HAdv-41, norovirus I and II, sapovirus, ASV, and EV present in stool, not meconium, of 6 asymptomatic infants; not maternally derived
Cheng et al., 2021 [55]	Age-matched infants with (*n* = 51) and without (*n* = 39) NEC	PCR/gel electrophoresis of stool	N/A	ASV most common; HAdv and EBV may be related to NEC severity; EV and HBoV only detected in NEC; RVA ↑ in controls
Kaelin et al., 2022 [56]	Preterm infants (< 27 wk; ≤ 1500 g) with (*n* = 9) or without (*n* = 14) NEC, collected longitudinally	Metagenomic sequencing of stool	MetaSPAdes and minimus2; tBLASTx queries of Gut Phage and Gut Virome databases	Convergence of viral signature with low β-diversity before NEC development; large interindividual variation
LaTuga et al., 2011 [85]	ELBW infants (*n* = 2; <1000 g, 25 wk)	Shotgun metagenomic sequencing of MDA-derived total genomic stool DNA	MG-RAST; SEED database	ssDNA phages (S13, phiX174, alpha 3), dsDNA phage (*Staphylococcus* Phage K), and HAdv C identified
Olm et al., 2019 [111]	Preterm infants with (*n* = 34) and without (*n* = 126) NEC	Genome-resolved metagenomics of stool	VICA, VirSorter, VirFinder, and vFam	NEC may be associated with increased phages compared to controls

DOL, days of life; DNA, deoxyribonucleic acid; CMV, cytomegalovirus; PCR, polymerase chain reaction; RNA, ribonucleic acid; MEGA7, molecular evolutionary genetics analysis version 7; RVA, rotavirus A; HAdv, human adenovirus; ASV, astrovirus; EV, enterovirus; NEC, necrotizing enterocolitis; EBV, Epstein-Barr virus; HBoV, human bocavirus; BLAST, nucleotide basic local alignment search tool; ELBW, extremely low birthweight infants; MDA, multiple displacement amplification; MG-RAST, metagenomic rapid annotations using subsystems technology.

## 8. Viral Involvement in Preterm Intestinal Inflammation

Enteric viruses have been detected in preterm infants at the time of NEC development [226,227,228,229], and several, including Coxsackie B2, CMV, and *Staphylococcus* phage 363_30 [230,231,232,233,234,235], have been associated, in isolation, with disease development. However, despite differences in prevalence of some enteric viruses in NEC and control infants [55], no causal role for enteric viruses has yet been identified in NEC pathogenesis [236,237]. Interestingly, CMV is both present in a small percentage of NEC cases [238], but also may present similarly to NEC, with diarrhea, fever, and generalized intestinal inflammation [239,240]. The only study to date evaluating the complete preterm virome, as well as interactions with the bacterial microbiome, indicates the gut virome of preterm infants with NEC is altered, along with the bacterial microbiome, in the 10 d preceding development of the disease [56]. Viral β-diversity decreases and converges upon a common signature in NEC infants, driven largely by changes in viruses of low abundance. In addition, viruses driving the convergence of diversity in NEC infants interacted uniquely with bacterial changes during this period [56]. As bacteriophage changes are evident in related gastrointestinal diseases (e.g., inflammatory bowel disease [IBD]) [241,242,243], these changes potentially indicate a role for the gut virome in neonatal NEC development. Further studies are required to elucidate whether these changes are causative in NEC pathogenesis.

The possibility of introducing viruses or propages to benefit neonatal intestinal inflammation, however, also exists. Brunse et al. [244] treated preterm piglets subjected to NEC with an oro-gastric fecal filtrate transplant (FFT), filtering out bacteria but allowing transfer of bacteria-associated phages. Interestingly, FFT increased abundance of mucosa-associated phages, particularly enterobacteria-infecting *Microviridae*, and was protective without subjecting piglets to risk of sepsis or activation of host mucosal immunity [244]. Of importance, FFT treatment did not increase the relative abundance of eukaryotic viruses capable of infecting mammalian cells.

## 9. Conclusions

Studies are slowly accumulating (Table 1 and Table 2) on the significant roles of fungi and viruses, and their interkingdom interactions, in preterm infant intestinal health and disease, but progress has been limited, given that most infant microbiome studies focus exclusively on the abundance, diversity, and function of the bacterial constituents. In order to provide a more inclusive and comprehensive analysis of the developing infant intestinal ecosystem, databases of nonmicrobial reference genomes need to be expanded in order to correctly identify microbial DNA and RNA sequences. In addition, methods for the identification of sequences should converge on the most precise, reliable, and consistent best-practice techniques in order to provide comparable information across studies. Finally, as fungi and viruses clearly impact preterm intestinal development and disease, precision medicine applications for diagnosis or therapy in these populations must consider the inclusion, and impact on, nonbacterial components of the microbiome, and in doing so, will likely maximize success in improving intestinal health outcomes among preterm infants.

## Data Availability

Not applicable.
